# Association of NLR with all-cause and cardiovascular mortality in adults with coronary heart disease: 1999–2018 NHANES data analysis

**DOI:** 10.1097/MD.0000000000040844

**Published:** 2024-12-13

**Authors:** Qian Chen, Xiao-Wei Dai, Qi-Qi Dong, Xin-Xin Zhang, Wen-Ting Ma

**Affiliations:** a Department of Geriatrics, Mengcheng First People’s Hospital, Mengcheng, Anhui, China.

**Keywords:** all-cause mortality, cardiovascular mortality, CHD, NHANES, NLR

## Abstract

The neutrophil-to-lymphocyte ratio (NLR) is an important inflammatory marker. However, the relationship between NLR and the prognosis of patients with coronary heart disease (CHD) remains unclear. The purpose of this study is to explore the relationship between NLR and all-cause mortality and cardiovascular mortality in CHD patients. This study analyzed data from 1625 CHD patients who participated in the National Health and Nutrition Examination Survey from 1999 to 2018. Multivariate Cox regression analysis was used to explore the relationship between mortality risk and NLR. The optimal NLR cutoff value related to survival outcomes was determined using the maximum selected rank method. Restricted cubic spline analysis was performed to investigate the correlation between NLR and mortality risk in CHD patients. Moreover, subgroup analyses were conducted to assess the relationship between NLR and all-cause and cardiovascular mortality in different populations. Additionally, time-dependent receiver operating characteristic curves were used to evaluate the accuracy of NLR in predicting survival outcomes. During a median follow-up of 88 months, a total of 475 patients experienced all-cause mortality, and 278 patients experienced cardiovascular mortality. After adjusting for confounding factors, compared with CHD patients with higher NLR, those with lower NLR had a 43% reduced risk of all-cause mortality (hazard ratio: 0.57, 95% CI: 0.41–0.8) and a 51% reduced risk of cardiovascular mortality (hazard ratio: 0.49, 95% CI: 0.3–0.78). Kaplan–Meier analysis showed that the survival rate in the high NLR group was significantly lower in terms of all-cause and cardiovascular mortality rates than in the low NLR group (*P* < .0001). The results of the restricted cubic spline analysis indicated a nonlinear relationship between NLR and all-cause mortality as well as cardiovascular mortality in CHD patients. In addition, receiver operating characteristic analysis showed that the area under the curve for all-cause mortality at 3 years, 5 years, and 10 years were 0.596, 0.591, and 0.604, while the area under the curve for cardiovascular mortality were 0.623, 0.617, and 0.623, in CHD patients. Elevated NLR is associated with increased risk of cardiovascular and all-cause mortality in CHD patients, and NLR can independently predict the prognosis of CHD patients.

## 1. Introduction

Cardiovascular disease is one of the most prevalent and deadly diseases worldwide, with complex pathogenesis. Research indicates that inflammation and oxidative stress are closely related to the pathogenesis of cardiovascular disease, and inflammatory biomarkers have become one of the current research hotspots.^[1]^ Among them, coronary heart disease (CHD), as one of the leading causes of death worldwide, is primarily associated with atherosclerosis. Atherosclerosis is a systemic, lipid-driven inflammatory disease in which chronic inflammation plays a crucial role in the formation and development of plaques.^[2]^ Therefore, the role of inflammatory biomarkers in the pathogenesis of atherosclerosis and CHD has become a highly researched field of interest.

The neutrophil-to-lymphocyte ratio (NLR) is a novel inflammatory biomarker that combines the counts of neutrophils and lymphocytes, 2 subtypes of white blood cells. An increase in the number of neutrophils is closely associated with inflammation, while a decrease in the number of lymphocytes is linked to stress response. Therefore, NLR can reflect the balance between inflammatory and immune responses, and it has significant clinical implications.^[3]^ Some scholars believe that compared with the count of neutrophils or lymphocytes alone, NLR is better able to predict the development of cardiovascular diseases,^[4]^ such as hypertension, heart failure, infective endocarditis, stable CHD, and acute coronary syndrome.^[5–9]^ However, although NLR may be an effective indicator, more rigorous research is needed to clarify the relationship between NLR and the risk of mortality in CHD patients.

The aim of this study is to investigate in-depth the prognostic value of the NLR in patients with CHD. Specifically, the research aims to explore the association between NLR and the risks of all-cause mortality and cardiovascular mortality in CHD patients, in order to provide reliable scientific support for predicting the development of CHD and developing more effective treatment plans.

## 2. Methods

### 2.1. Study population

**Figure 1.**
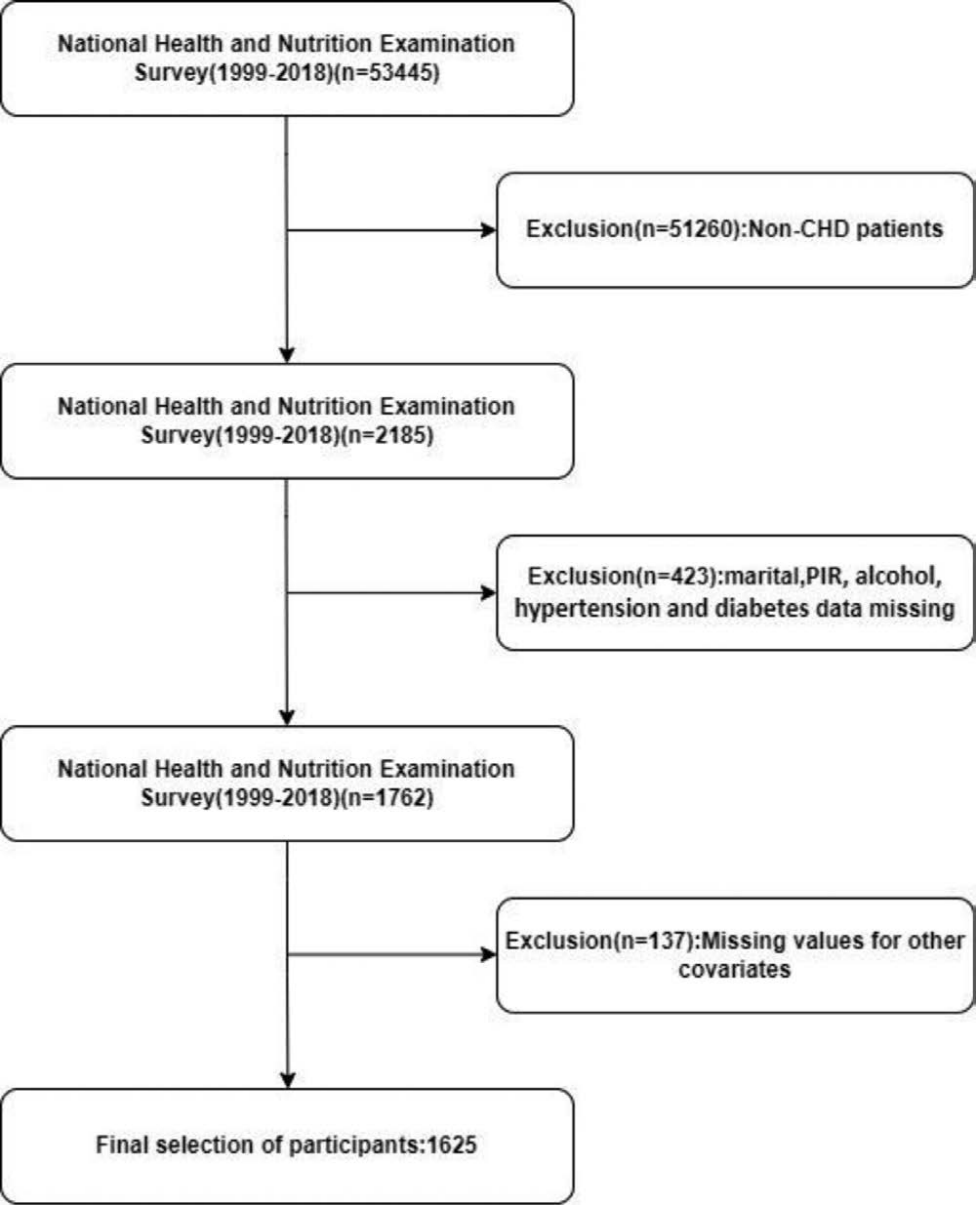
Study flow chart.

To ensure the reliability of the study results, sample screening was carried out according to the following exclusion criteria in this study: (1) individuals aged under 18; (2) individuals with missing data on neutrophils, lymphocytes, and mortality information. In addition, individuals who lacked information on study variables such as education level, marital status, poverty-income ratio, body mass index (BMI), and laboratory data were also excluded. Finally, a total of 1625 eligible patients with CHD were selected for the study. The detailed research process is shown in Figure 1. It is worth mentioning that due to the openness and originality of the National Health and Nutrition Examination Survey (NHANES) data, our study was granted exemption from the ethics committee, and therefore, ethical approval was not required.

### 2.2. Definition of CHD

In this study, we used the history of CHD to determine the subjects.^[10]^ Specifically, in the health survey questionnaire, we asked participants if they had ever been told by a doctor or other health professional that they had CHD. If the answer was “yes,” they were classified as having CHD. This method is effective in diagnosing CHD and ensures the accuracy of the results.

### 2.3. Death related information

The main outcomes of this study include all-cause mortality and cardiovascular disease mortality. To determine the patients’ mortality status, we correlated the mortality information from NHANES between 2003 and 2018 with the death rate data from the National Death Index death certificate records, and used a probability matching algorithm for matching. For cardiovascular mortality, we estimated it based on coding in the International Classification of Diseases, Tenth Revision (ICD-10), including I00 to I09, I11, I13, I20 to I51, and I60 to I69 codes. Finally, we conducted long-term follow-up until the patient’s death or December 31, 2019, to obtain reliable study conclusions.

### 2.4. NLR

In this study, we used the Beckman Coulter automatic blood analyzer to perform a complete blood cell count and obtain the absolute counts of neutrophils and lymphocytes, which were expressed as ×-10^3^ cells/µL. We then calculated the NLR using the method of dividing the absolute neutrophil count by the absolute lymphocyte count,^[11]^ to evaluate the patients’ immune status and degree of inflammation.

### 2.5. Covariates

To account for potential factors that may affect CHD mortality, we included multiple covariates in this study. These covariates included age, gender, race, education level, marital status, poverty-income ratio, diabetes, hypertension, BMI, as well as information related to smoking and drinking. These factors have been previously verified by research and clinical experience to be associated with CHD mortality, thus we considered and controlled for them in our analysis. This can effectively improve the reliability and accuracy of our study conclusions.

In addition, laboratory examinations were performed on individuals in this study, including the following tests: albumin, estimated glomerular filtration rate (eGFR), hemoglobin, glycated hemoglobin, uric acid, and blood urea nitrogen (BUN).

### 2.6. Statistical analysis

**Figure 2.**
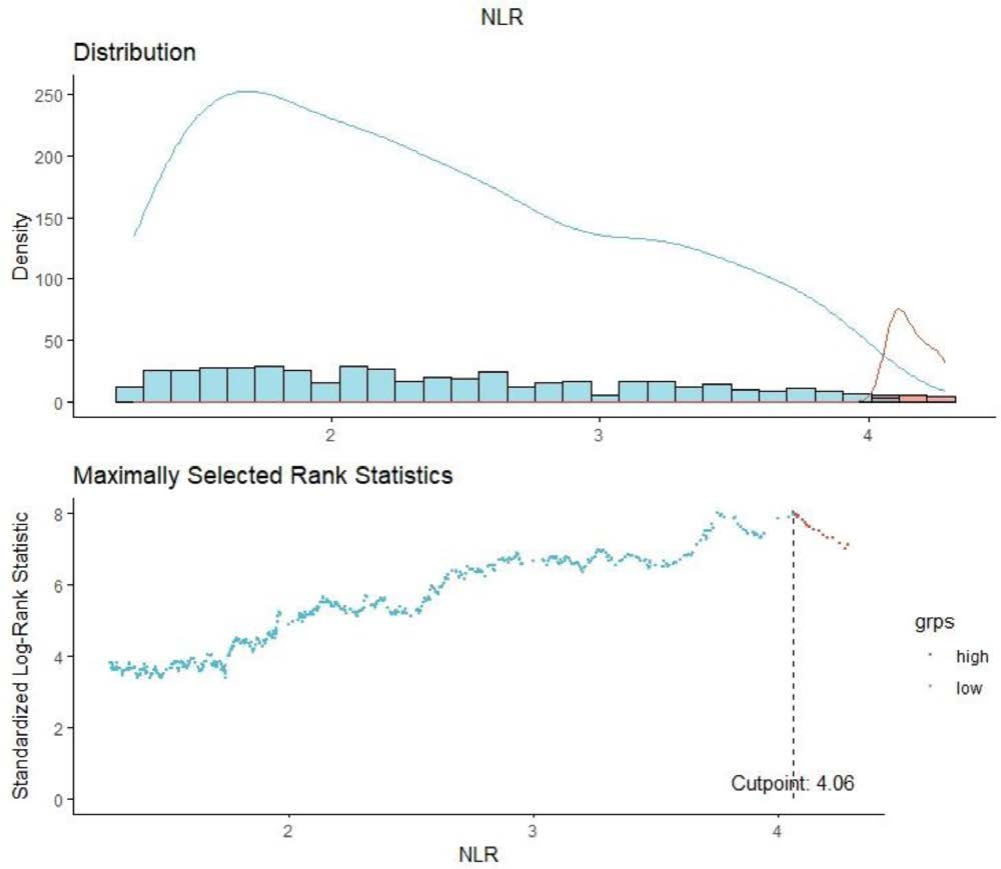
Determination of the NLR cutoff point using maximally selected rank statistics. Standardized log-rank statistic was utilized in the calculation. NLR = neutrophil-to-lymphocyte ratio.

R software (version 4.3.2, https://www.r-project.org) was used for data analysis in this study. The optimal NLR cutoff point that was most significantly correlated with survival outcomes was determined by using the Maximum Selected Rank Statistics method from the “maxstat” package. Participants were then divided into high and low NLR groups based on this cutoff point, as shown in Figure 2.

Continuous variables are presented as means (SE) and categorical variables are presented as frequencies (%). In this study, a multivariate Cox proportional hazards regression model was used to explore the relationship between NLR and the risk of all-cause and cardiovascular mortality in patients with CHD. Three models were established: Model 1 was unadjusted, Model 2 was adjusted for age, race, and gender, and Model 3 was further adjusted for smoking, drinking, hypertension, diabetes, eGFR, uric acid, and BUN on the basis of Model 2.

In this study, the Kaplan–Meier method was used for survival analysis and the log-rank test was used to compare the survival probabilities of CHD patients at different NLR levels. In addition, stratified analyses were conducted for factors such as age, gender, race, BMI, hypertension, and diabetes. Furthermore, the relationship between NLR and the risk of all-cause and cardiovascular mortality in CHD patients was explored using the restricted cubic spline (RCS) method. The “timeROC” package was used to assess the accuracy of NLR in predicting survival outcomes at different time points.^[12]^ Results were considered statistically significant when *P* < .05 (two-tailed).

## 3. Results

### 3.1. Baseline characteristics of participants

A total of 1625 CHD patients were finally included in this study and were divided into high and low NLR groups. Compared with the low NLR group, patients in the high NLR group were older, had a higher proportion of males and non-Hispanic Whites, lower levels of albumin and eGFR, while their values of creatinine, uric acid, and BUN were higher (see Table 1 for details).

**Table 1:** Basic characteristics of participates with CHD based on NLR category.

Variables	Total	High	Low	*P*-value
Age (years, mean (SE))	66.55 (0.36)	69.65 (0.97)	66.12 (0.38)	**<.001**
HbA1c (%)	6.13 (0.04)	6.10 (0.11)	6.14 (0.04)	.77
Albumin (g/dL)	4.19 (0.01)	4.12 (0.02)	4.20 (0.01)	**.003**
eGFR (mL/min/1.73 m^2^)	71.75 (0.66)	66.34 (1.81)	72.50 (0.69)	**.002**
Creatinine (µmol/L, mean (SE))	96.42 (1.43)	101.31 (2.70)	95.74 (1.55)	.07
Uric acid (µmol/L, mean (SE))	361.96 (3.20)	380.51 (9.49)	359.41 (3.25)	**.03**
BUN (mmol/L, mean (SE) )	6.39 (0.10)	7.32 (0.30)	6.27 (0.10)	**.001**
Hemoglobin (g/dL, mean (SE) )	14.18 (0.05)	14.02 (0.18)	14.20 (0.05)	.31
Platelets (×10^9^/L, mean (SE))	223.27 (1.96)	223.10 (6.77)	223.29 (2.02)	.98
BMI n (%)				.82
<25	20.05 (0.02)	21.79 (3.22)	19.81 (1.26)	
25–30	34.78 (0.02)	33.07 (4.19)	35.02 (1.67)	
>30	45.16 (0.03)	45.14 (4.71)	45.17 (1.79)	
Sex n (%)				.08
Male	65.82 (0.03)	72.49 (3.7)	64.91 (1.7)	
Female	34.18 (0.02)	27.51 (3.7)	35.09 (1.7)	
Race n (%)				.05
Mexican American	3.16 (0)	2.16 (0.7)	3.3 (0.5)	
Non-Hispanic Black	5.81 (0)	1.89 (0.83)	6.35 (0.56)	
Non-Hispanic White	83.37 (0.04)	89.23 (2.39)	82.57 (1.24)	
Other	7.66 (0.01)	6.73 (2.29)	7.79 (0.97)	
Marital n (%)				.3
Married	64.74 (0.04)	60.44 (3.8)	65.33 (1.82)	
Never married	4.57 (0.01)	6.92 (2.29)	4.25 (0.8)	
Divorced	10.33 (0.01)	8.78 (2.27)	10.54 (1.03)	
Unmarried but have/had partner	20.36 (0.02)	23.86 (3.2)	19.88 (1.44)	
Education n (%)				.92
Less than high school	24.35 (0.02)	23.07 (3.34)	24.53 (1.46)	
High school or equivalent	26.1 (0.02)	26.85 (3.89)	26 (1.68)	
College or above	49.55 (0.03)	50.08 (3.79)	49.48 (1.83)	
Smoke n (%)				.3
Never	34.41 (0.02)	27.97 (3.84)	35.29 (1.67)	
Former	47.71 (0.03)	52.88 (4.62)	47 (1.67)	
Now	17.88 (0.02)	19.15 (3.82)	17.7 (1.48)	
Alcohol (n (%))				.08
Never	10.77 (0.01)	10.3 (2.63)	10.84 (0.96)	
Former	29.07 (0.02)	39.22 (4.15)	27.67 (1.64)	
Mild	42.53 (0.03)	36.41 (3.92)	43.37 (1.98)	
Moderate	8.42 (0.01)	6.45 (2.11)	8.69 (0.97)	
Heavy	9.21 (0.01)	7.62 (2.49)	9.43 (1.02)	
Diabetes (n (%))				.12
Yes	39.75 (0.02)	42 (3.83)	39.33 (1.6)	
No	49.46 (0.03)	42.76 (4.38)	50.38 (1.65)	
Borderline	10.8 (0.01)	15.23 (3.23)	10.18 (0.98)	
Hypertension (n (%))				.63
Yes	75.73 (0.04)	77.6 (3.93)	75.48 (1.51)	
No	24.27 (0.02)	22.4 (3.93)	24.52 (1.51)	
PIR (n (%))				.48
<1	11.3 (0.01)	9.33 (2.06)	11.57 (1.03)	
1–3	42.63 (0.03)	46.7 (4.52)	42.07 (1.79)	
>3	46.07 (0.03)	43.97 (4.68)	46.36 (1.99)	

Date are presented as mean (SE) or n (%). Bold values are indicate *P* < .05.

BMI = body mass index, BUN = blood urea nitrogen, CHD = coronary heart disease, eGFR = estimated glomerular filtration rate, HbA1c = glycosylated hemoglobin, NLR = neutrophil-to-lymphocyte ratio, PIR = poverty-income ratio.

### 3.2. Association of NLR with all-cause mortality in patients with CHD

**Figure 3.**
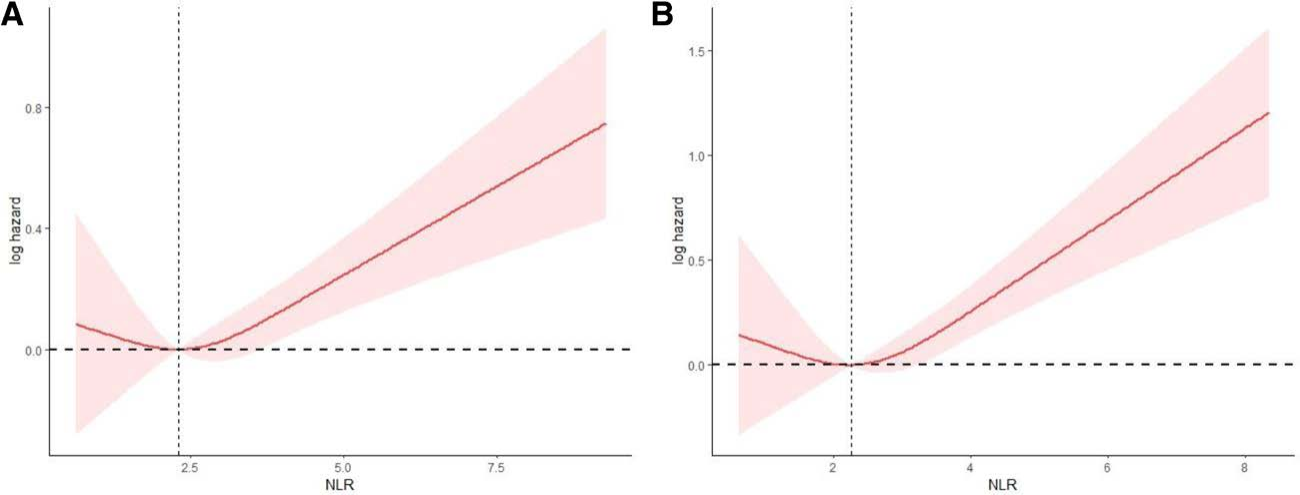
Restricted cubic spline (RCS) illustrating the relationship between NLR and all-cause mortality (A) and cardiovascular mortality (B) among participates with CHD. CHD = coronary heart disease, NLR = neutrophil-to-lymphocyte ratio.

During the follow-up of 1625 CHD patients, 475 patients died from various causes. As shown in Table 2, in Model 1, there was a significant association between NLR and the risk of all-cause mortality (hazard ratio [HR]: 1.17, 95% CI: 1.11–1.24). After adjusting for multiple variables, the risk of all-cause mortality significantly increased with each unit increase of NLR. Model 2 (HR: 1.13, 95% CI: 1.07–1.19) and Model 3 (HR: 1.1, 95% CI: 1.05–1.16) showed an increase of 13% and 10%, respectively. RCS analysis revealed a nonlinear relationship between NLR and the risk of all-cause mortality in CHD patients, as shown in Figure 3A.

**Table 2:** Association of NLR with mortality risk with CHD.

Variables	Model 1		Model 2		Model 3	
	HR (95% CI)	*P*	HR (95% CI)	*P*	HR (95% CI)	*P*
*Cardiovascular mortality*						
NLR	1.24 (1.11, 1.38)	**<.001**	1.22 (1.11, 1.35)	**<.0001**	1.2 (1.07, 1.34)	**.002**
NLR category						
** **Higher NLR	Ref	Ref	Ref	Ref	Ref	Ref
** **Lower NLR	0.29 (0.19, 0.44)	**<.0001**	0.41 (0.27, 0.62)	**<.0001**	0.49 (0.3, 0.78)	**.003**
*All-cause mortality*						
NLR	1.17 (1.11, 1.24)	**<.0001**	1.13 (1.07, 1.19)	**<.0001**	1.1 (1.05, 1.16)	**<.001**
NLR category						
** **Higher NLR	Ref	Ref	Ref	Ref	Ref	Ref
** **Lower NLR	0.4 (0.29, 0.55)	**<.0001**	0.5 (0.36, 0.69)	**<.0001**	0.57 (0.41, 0.8)	**.001**

Model 1: no adjustments made; Model 2: adjusted for age, sex, eth; Model 3: adjusted for age, sex, eth, smoke, alcohol, hypertension, diabetes, eGFR, UA, BUN. Bold values are indicate *P* < .05.

BUN = blood urea nitrogen, CHD = coronary heart disease, CI = confidence interval, eGFR = estimated glomerular filtration rate, HR = hazard ratio, NLR = neutrophil-to-lymphocyte ratio, Ref = reference, UA = uric acid.

**Figure 4.**
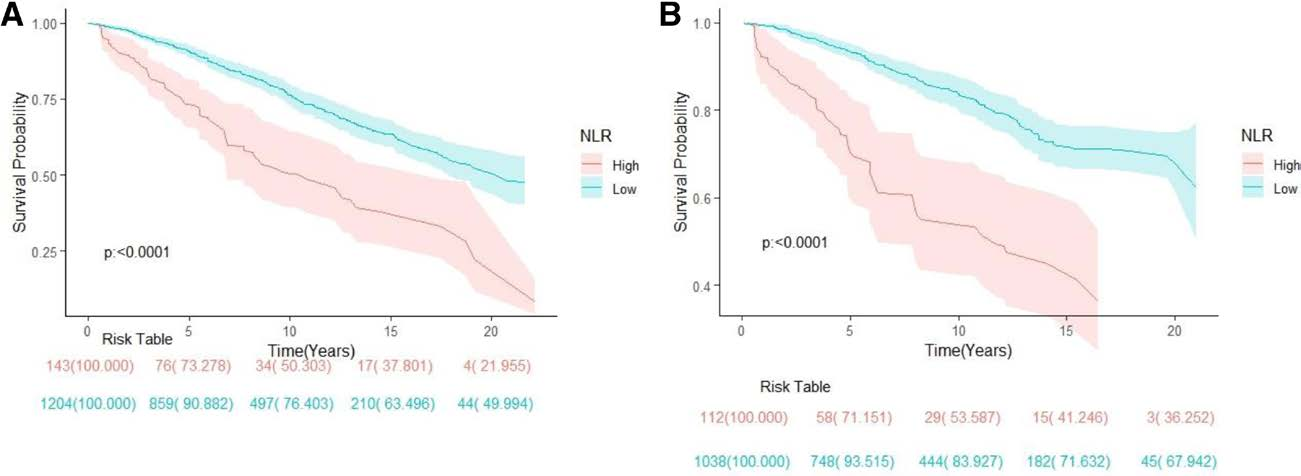
Kaplan–Meier curves of all-cause mortality (A) and cardiovascular mortality (B) among participates with CHD. CHD = coronary heart disease.

The survival analysis conducted in this study showed a significant decrease in survival rate for the high NLR group compared to the low NLR group (*P* < .0001), (as shown in Fig. 4A). Each unit decrease in NLR was associated with a 50% reduction in the risk of all-cause mortality in different multivariate models (Model 2: HR: 0.5, 95% CI: 0.36–0.69, and Model 3: HR: 0.57, 95% CI: 0.41–0.8) as shown in Table 2. Subgroup analysis revealed that the association between NLR and all-cause mortality was not consistent in BMI stratification (interaction *P* < .05) after stratification by age, gender, ethnicity, BMI, smoking status, hypertension, and diabetes. Specifically, among CHD patients with higher BMI, lower NLR was associated with lower risk of all-cause mortality (see Table 3 for details).

**Table 3:** Subgroup analyses of NLR and mortality risk in CHD.

	Cardiovascular mortality		*P* for interaction	All-cause mortality		*P* for interaction
	Higher NLR	Lower NLR		Lower NLR	Lower NLR	
		HR (95% CI)			HR (95% CI)	
Age			.14			.1
** **<65 = 2	Ref	0.17 (0.06, 0.5)		Ref	0.3 (0.18, 0.52)	
** **≥65 = 1	Ref	0.46 (0.29, 0.73)		Ref	0.58 (0.4, 0.84)	
Sex			.32			.11
** **Male = 0	Ref	0.65 (0.19, 2.23)		Ref	0.41 (0.24, 0.71)	
** **Female = 1	Ref	0.38 (0.22, 0.63)		Ref	0.63 (0.41, 0.96)	
Race			.12			.37
** **Mexican American = 4	Ref	1.56 (0.15, 16.04)		Ref	0.07 (0.03, 0.21)	
** **Non-Hispanic Black = 3	Ref	0 (0, 0.04)		Ref	0.25 (0.06, 1.01)	
** **Non-Hispanic White = 2	Ref	0.46 (0.28, 0.74)		Ref	0.57 (0.4, 0.8)	
** **Other = 1	Ref	e^-02^(0, 1.7e^-01^)		Ref	0.13 (0.02, 0.73)	
BMI			.28			**.04**
** **<25	Ref	0.53 (0.15, 1.91)		Ref	1.45 (0.55, 3.85)	
** **25–30	Ref	0.34 (0.16, 0.72)		Ref	0.32 (0.19, 0.52)	
** **>30	Ref	0.44 (0.18, 1.04)		Ref	0.5 (0.27, 0.93)	
Hypertension			**<.001**			.94
** **Yes	Ref	0.37 (0.23, 0.62)		Ref	0.56 (0.38, 0.83)	
** **No	Ref	3.28 (0.95, 11.28)		Ref	0.54 (0.27, 1.08)	
Diabetes			.88			.31
** **Yes	Ref	0.45 (0.21, 0.97)		Ref	0.37 (0.25, 0.55)	
** **No	Ref	0.45 (0.23, 0.91)		Ref	0.84 (0.43, 1.63)	
** **Borderline	Ref	0.08 (0.02, 0.39)		Ref	0.43 (0.18, 1.03)	

Adjusted for age, sex, eth, smoke, alcohol, hypertension, diabetes, eGFR, UA, BUN. Bold values are indicate *P* < .05.

CHD = coronary heart disease, CI = confidence interval, HR = hazard ratio, NLR = neutrophil-to-lymphocyte ratio, Ref = reference, UA = uric acid.

### 3.3. Association between NLR and cardiovascular death in patients with CHD

During the follow-up of 1625 CHD patients, 278 patients died from cardiovascular diseases. RCS analysis showed a nonlinear relationship between NLR and cardiovascular mortality in CHD patients (as shown in Fig. 3B). In Model 1, there was a significant association between NLR and the risk of cardiovascular disease (HR: 1.24, 95% CI: 1.11–1.38). After adjusting for multiple variables, the risk of cardiovascular mortality increased by 22% and 20% for each unit increase of NLR in Model 2 (HR: 1.22, 95% CI: 1.11–1.35) and Model 3 (HR: 1.2, 95% CI: 1.07–1.34), respectively (see Table 2 for details).

The survival analysis also revealed a significant decrease in survival rate for the high NLR group compared to the low NLR group (*P* < .0001) in CHD patients with cardiovascular mortality (as shown in Fig. 4B). Cox regression analysis confirmed a significant reduction in cardiovascular mortality for the low NLR group compared to the high NLR group. In different multivariate models, the risk of cardiovascular mortality decreased by 71% (Model 1: HR: 0.29, 95% CI: 0.19–0.44), 59% (Model 2: HR: 0.41, 95% CI: 0.27–0.62), and 51% (Model 3: HR: 0.49, 95% CI: 0.3–0.78), respectively. Subgroup analysis explored the relationship between NLR and cardiovascular mortality. The results showed that the association between NLR and cardiovascular mortality was not consistent in hypertension stratification (interaction *P* < .05). Specifically, for CHD patients with comorbid hypertension, lower NLR was associated with lower risk of cardiovascular mortality (see Table 2 for details).

### 3.4. Prognostic value of NLR in patients with CHD

**Figure 5.**
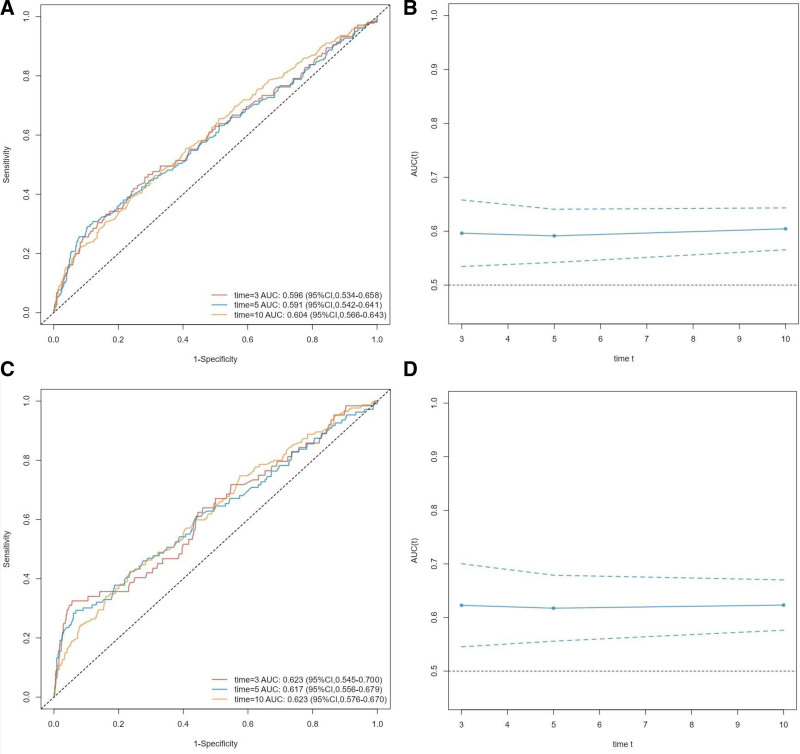
Time-dependent ROC curves and time-dependent AUC values (with 95% confidence band) of the NLR for predicting all-cause mortality (A and B) and cardiovascular mortality (C and D). AUC = area under the curve, ROC = receiver operating characteristic, NLR = neutrophil-to-lymphocyte ratio.

According to receiver operating characteristic analysis, NLR showed similar predictive ability for all-cause mortality over 3 years, 5 years, and 10 years with area under the curves of 0.596, 0.591, and 0.604, respectively (as shown in Fig. 5A and B). Similarly, NLR also showed similar predictive ability for cardiovascular mortality over 3 years, 5 years, and 10 years with area under the curves of 0.623, 0.617, and 0.623, respectively (as shown in Fig. 5C and D). These results suggest that NLR has effective predictive ability for mortality over different time periods.

## 4. Discussion

This study is the first to use the NHANES database to reveal a nonlinear positive correlation between NLR and all-cause mortality and cardiovascular mortality risk in adult CHD patients in the United States. The results of the study indicate a significant increase in the risk of all-cause mortality and cardiovascular mortality associated with an increase in NLR in CHD patients. Subgroup analyses found that an increase in BMI was associated with a significant increase in the risk of all-cause mortality in CHD patients, while CHD patients with comorbid hypertension had a significantly increased risk of cardiovascular mortality. Moreover, NLR was found to have effective predictive ability for all-cause mortality and cardiovascular mortality risk over 3 years, 5 years, and 10 years.

CHD is currently one of the most common cardiovascular diseases, and its incidence and mortality rates continue to rise. In developed countries, CHD is the leading cause of death, while in developing countries it ranks second. Every year, millions of people worldwide die from CHD and its complications, with low-income developing countries being the most affected.^[13]^ In recent years, a broad range of scholars and clinicians have recognized the importance of primary prevention of CHD. Multiple studies have shown that the occurrence of CHD is the result of the combined effects of various traditional and novel risk factors. Risk factor control plays an indispensable role in primary prevention of CHD. Traditional risk factors have received clinical research attention and are actively treated. Meanwhile, increasing basic and clinical research, as well as various epidemiological data, indicate that some new risk factors such as NLR, blood uric acid levels, platelet-to-lymphocyte ratio, red blood cell distribution width, CRP, HCY, etc, play important roles in the occurrence and development of CHD, providing new basis for CHD prevention.^[14]^

The pathological basis of CHD is atherosclerosis of the coronary arteries, which is a slow and complex pathological process involving multiple factors such as endothelial injury, lipid deposition, platelet aggregation, infiltration of inflammatory cells, oxidative stress, and smooth muscle proliferation and migration.^[15]^ Studies have shown that atherosclerosis is not just a simple process of lipid deposition, and that inflammatory reactions play a key role in the formation, progression, and rupture of atherosclerotic plaques. The reduced stability of atherosclerotic plaques, rupture, vasospasm, and the resulting secondary platelet adhesion, aggregation, and thrombus formation are the main pathological and physiological mechanisms underlying acute myocardial infarction.^[16]^ In the pathogenesis of atherosclerosis, inflammation plays a key role.^[17]^ The higher the inflammatory markers, the higher the degree of coronary artery lesions and the rate of cardiovascular death in CHD patients. Clinical studies have shown that white blood cell count, lymphocyte count, neutrophil count are closely related to CHD.^[18]^ Many basic research studies have shown that after acute myocardial infarction, a large number of white blood cells infiltrate into the ischemic area, the deformability of white blood cells decreases, and they move more slowly in capillaries, making it more likely to cause capillary blockage in the ischemic area, thereby exacerbating myocardial microcirculation disorders.^[19]^ In this process, neutrophils can aggravate myocardial damage by releasing a large amount of acid phosphatase, oxygen free radicals, peroxidase, or promoting the aggregation of white blood cells and platelets, leading to cardiogenic shock, malignant arrhythmia, and sudden death.^[20]^ In addition, numerous clinical studies have shown that there is a certain correlation between white blood cell count, neutrophil count, monocyte count, and the occurrence and prognosis of CHD.^[21,22]^

In recent years, the role of NLR in predicting the prognosis of CHD has attracted increasing attention and become a hot spot in the study of the correlation between inflammatory markers and CHD. Neutrophils accelerate the progression of atherosclerotic plaques by activating nonspecific inflammation and releasing inflammatory factors. NLR represents 2 different and complementary immune system pathways, reflecting the balance between the anti-inflammatory component lymphocytes and the pro-inflammatory component neutrophils. Therefore, NLR has a higher predictive value in cardiovascular disease and its prognosis than other white blood cells and their subtypes, and is more effective.^[23]^ Studies have shown a correlation between NLR and chronic occlusive disease.^[24]^ Other studies have confirmed the correlation between NLR and the degree of coronary stenosis and prognosis in acute coronary syndrome,^[25]^ NLR being an independent predictor of long-term mortality after CHD interventional therapy,^[26]^ and the relationship between NLR and sudden cardiac death in patients with stable angina pectoris.^[27]^ In addition, some clinical studies by foreign scholars have found that an elevated NLR is an independent risk factor for the progression of atherosclerotic plaque lesions, recurrent stenosis of coronary stents, cardiogenic death after PCI or CABG surgery, and long-term poor prognosis in ACS patients.^[28–32]^ In conclusion, most studies consistently indicate that NLR is a risk factor for the short-term and long-term prognosis of acute coronary syndrome in CHD patients, NLR is expected to be a predictor of risk stratification in patients with diabetes.

In this study, our findings showed significant differences in all-cause and cardiovascular mortality between the high and low NLR groups when NLR was used as a categorical variable (Fig. 4 and Table 2). The optimal threshold (4.06) is defined by the maximum choice rank statistic, an outcome-oriented technique that provides a cutoff point corresponding to the most significant association with survival outcome. Our study showed that elevated NLR in CHD patients was associated with all-cause mortality and cardiovascular mortality after adjusting for confounders, whether grouped using maximum selected rank statistics or not. The underlying mechanism may be that increased neutrophils can aggravate chronic inflammation,^[33]^ and lymphopenia leads to decreased immune defense, which leads to decreased individual immunity and decreased resistance to disease.^[34]^

In addition, their predictive power for all-cause mortality and cardiovascular mortality risk over the next 3 years, 5 years, and 10 years was demonstrated using NLR values as continuous variables (Fig. 5). This suggests that neutrophils and lymphocytes, as key components, play a role in chronic inflammation and immune responses throughout the development of CHD.

This study found a nonlinear correlation between NLR and all-cause mortality and cardiovascular mortality in patients with CHD. The stratification results showed that the higher the BMI of CHD patients, the higher their risk of all-cause mortality, while the risk of cardiovascular mortality in CHD patients with hypertension also increased accordingly. Therefore, the results of this study suggest that controlling weight and blood pressure may help to reduce the mortality rate of CHD patients, but this needs to be further validated.

The main strengths of this study are that there was sufficient follow-up time, the conclusions were reliable, and sufficient statistical persuasion. However, there are still some limitations to this study. Firstly, despite our efforts to control various potential confounding factors, we cannot completely ignore other unknown variables that may affect NLR. Secondly, considering that this observational analysis excluded a large number of NHANES data with missing participants, there may be potential selection bias. Thirdly, although our study is based on the NHANES database, the sample size of CHD patients is not very large, and studies with large multicenter large sample sizes are needed for further validation at a later stage. Finally, our study results are based on a sample of Americans, so further exploration is needed to determine the feasibility of extending them to other populations.

## 5. Conclusions

In summary, we analyzed 1625 patients with CHD from the NHANES database and revealed the relationship between NLR and the risk of all-cause mortality and cardiovascular mortality during a long-term follow-up period. Our study results suggest that for patients with CHD, taking weight management and blood pressure intervention seriously is crucial in preventing, delaying, or reducing the progression of CHD and CHD-related death.

## Author contributions

**Conceptualization:** Qian Chen.

**Formal analysis:** Xiao-Wei Dai, Qi-Qi Dong.

**Methodology:** Xin-Xin Zhang, Wen-Ting Ma.

**Writing – original draft:** Qian Chen.

**Writing – review & editing:** Qian Chen.
